# Removal of obstructed pancreatic stent tube and biliary stones in patient with Roux-en-Y anatomy under direct endoscopic view

**DOI:** 10.1055/a-2368-4440

**Published:** 2024-08-07

**Authors:** Jingjing Yao, Shengxue Pan, Hongbo Li, Kunpeng Liu, Guangyao Zhao, Jindong Fu

**Affiliations:** 1 549615 Department of Gastroenterology, Rizhao People's Hospital, Rizhao, China; 2 549615 Department of General Surgery, Rizhao People's Hospital, Rizhao, China

A 42-year-old man was admitted due to upper abdominal pain for 2 hours. He had undergone a pancreaticoduodenectomy for a pancreatic tumor with a Roux-en-Y anastomosis 3 years earlier. Over the past year, he had experienced recurrent episodes of acute pancreatitis. Abdominal computed tomography scans revealed high-density images at the terminus of the pancreatic stent tube and the hilar bile duct ( Fig. 1 ). Stent obstruction was suspected and endoscopic removal was performed ( Video 1 ).

**Fig. 1:**
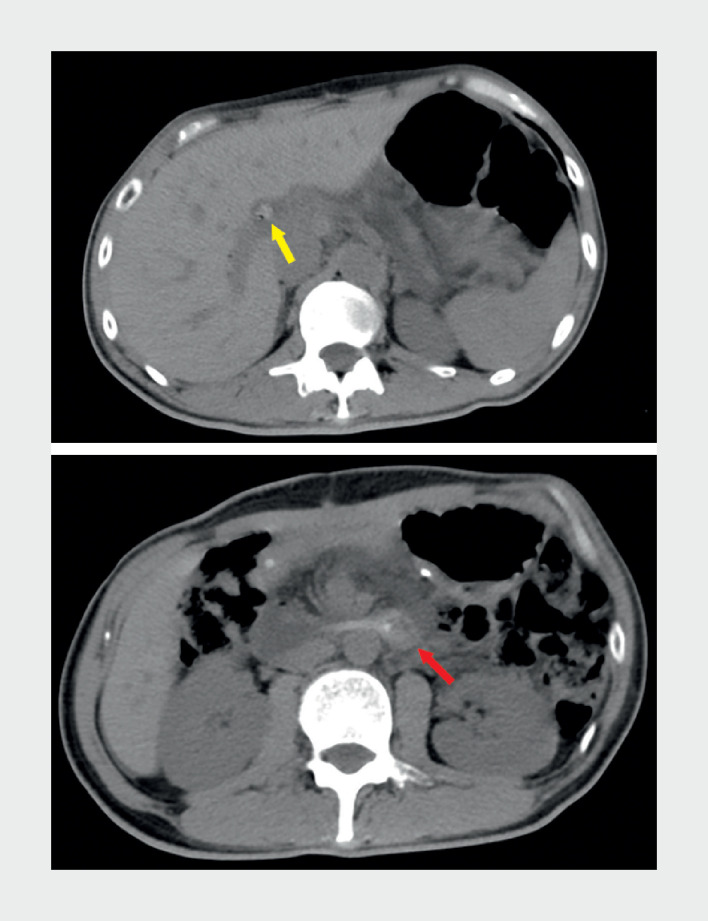
Abdominal computed tomography scans revealed high-density images at the terminus of the pancreatic stent tube (red arrow) and the hilar bile duct (yellow arrow).

Endoscopic removal of the obstructed pancreatic stent tube and biliary stones.Video 1

We inserted an Olympus PCF-260 enteroscope (Olympus, Tokyo, Japan) through the oral cavity into the jejunal input loop, reaching the pancreaticojejunal anastomosis site. Here we observed a long pancreatic stent tube affixed to the intestinal wall with sutures. An adhesive stone was found at the distal end, completely obstructing the lumen ( Fig. 2 ). Endoscopic scissors were employed to cut the sutures and the stent to facilitate stent removal. A snare device was then used to sequentially extract the stent and the attached stone. The choledochojejunal anastomosis was identified adjacent to the pancreatic anastomosis. Upon insertion of a cholangioscope (Eye-Max CDS11001, 9 Fr; Micro-Tech, Nanjing, China) for direct visualization ( Fig. 3 ), two calculi were revealed at the bile duct convergence ( Fig. 4 ). Following anastomosis dilation with a balloon catheter, the calculi were successfully extracted using a stone retrieval basket under direct visual guidance from the cholangioscope ( Video 1 ). Finally, a hemostatic clip was applied to constrict the dilated anastomosis, preventing reflux cholangitis.

**Fig. 2:**
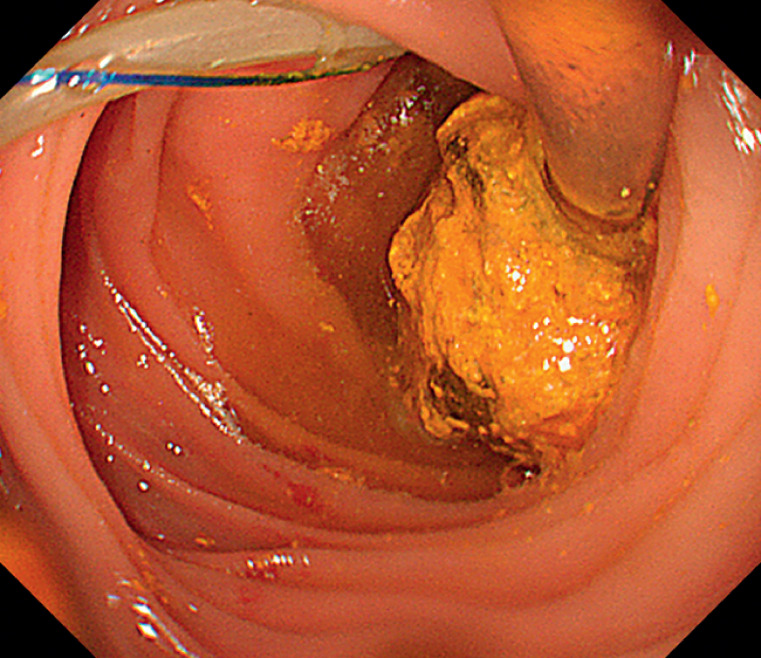
An adhesive stone was found at the distal end of the pancreatic stent tube, completely obstructing the lumen.

**Fig. 3:**
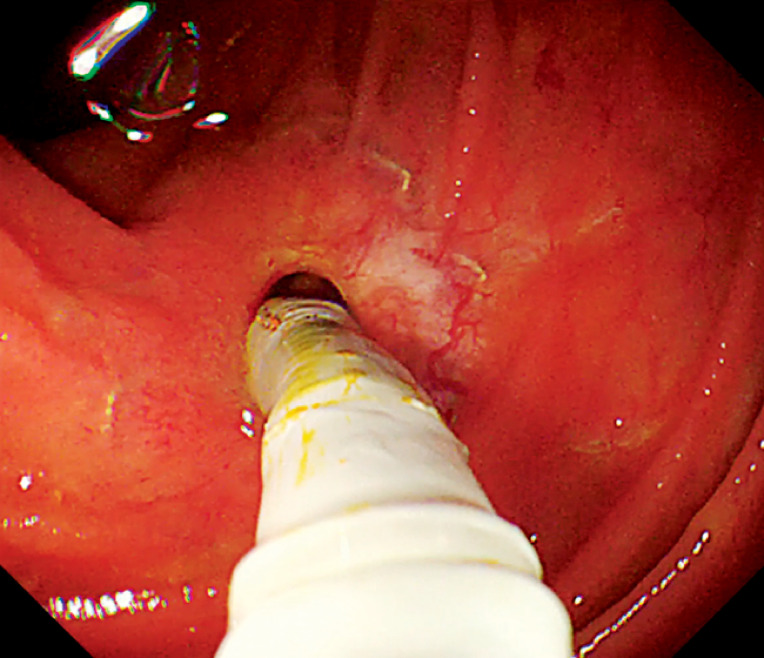
A cholangioscope was inserted into the bile duct through the choledochojejunal anastomosis.

**Fig. 4:**
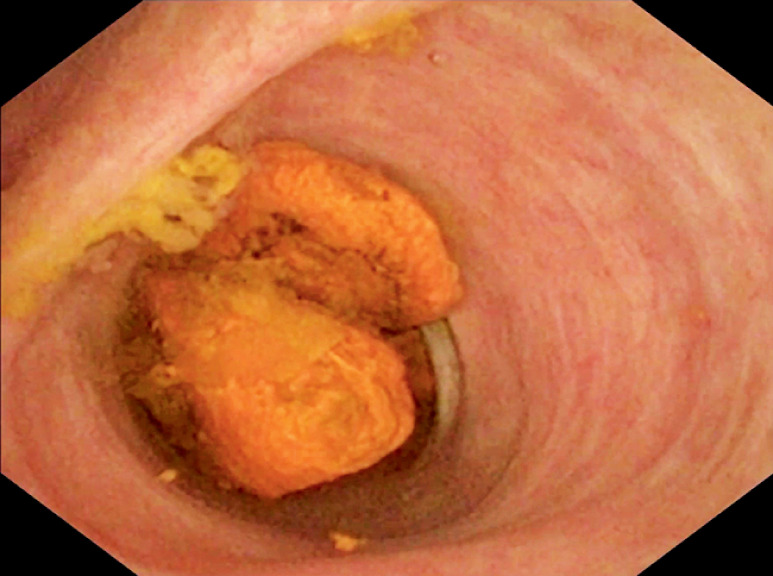
Two calculi were visible at the bile duct convergence under direct visualization with the cholangioscope.

The patient was kept fasting for 48 hours. No complications were reported postoperatively. His abdominal symptoms resolved. He was discharged 4 days postoperatively.

Following pancreatic surgery, stent tubes are commonly used to reduce the risk of pancreatic fistula formation 1 2 . In this rare case, the stent was completely obstructed by a stone, leading to recurrent episodes of acute pancreatitis. However, with the aid of endoscopic scissors, the obstructed stent was successfully removed endoscopically. Furthermore, for this patient with Roux-en-Y anatomy presenting with bile stones, the utilization of the cholangioscope was more intuitive and accurate, offering a direct and radiation-free approach.

Endoscopy_UCTN_Code_TTT_1AR_2AG

## Notes

Endoscopy E-Videos https://eref.thieme.de/e-videos
*E-Videos* is an open access online section of the journal *Endoscopy* , reporting on interesting cases and new techniques in gastroenterological endoscopy. All papers include a high-quality video and are published with a Creative Commons CC-BY license. Endoscopy E-Videos qualify for HINARI discounts and waivers and eligibility is automatically checked during the submission process. We grant 100% waivers to articles whose corresponding authors are based in Group A countries and 50% waivers to those who are based in Group B countries as classified by Research4Life (see: https://www.research4life.org/access/eligibility/ ). This section has its own submission website at https://mc.manuscriptcentral.com/e-videos .
